# Supported Ionic Liquids Used as Chromatographic Matrices in Bioseparation—An Overview

**DOI:** 10.3390/molecules27051618

**Published:** 2022-02-28

**Authors:** Sandra C. Bernardo, Rita Carapito, Márcia C. Neves, Mara G. Freire, Fani Sousa

**Affiliations:** 1 CICS-UBI—Health Sciences Research Centre, University of Beira Interior, Av. Infante D. Henrique, 6200-506 Covilhã, Portugal; sandracsbernardo@gmail.com (S.C.B.); ritacarapito@gmail.com (R.C.); 2 CICECO—Aveiro Institute of Materials, Chemistry Department, University of Aveiro, 3810-193 Aveiro, Portugal; mcneves@ua.pt

**Keywords:** biomolecules, chromatographic supports, ionic liquids, ligands immobilization, selectivity

## Abstract

Liquid chromatography plays a central role in biomanufacturing, and, apart from its use as a preparative purification strategy, either in biopharmaceuticals or in fine chemicals industries, it is also very useful as an analytical tool for monitoring, assessing, and characterizing diverse samples. The present review gives an overview of the progress of the chromatographic supports that have been used in the purification of high-value products (e.g., small molecules, organic compounds, proteins, and nucleic acids). Despite the diversity of currently available chromatographic matrices, the interest in innovative biomolecules emphasizes the need for novel, robust, and more efficient supports and ligands with improved selectivity. Accordingly, ionic liquids (ILs) have been investigated as novel ligands in chromatographic matrices. Given herein is an extensive review regarding the different immobilization strategies of ILs in several types of supports, namely in silica, Sepharose, and polymers. In addition to depicting their synthesis, the main application examples of these supports are also presented. The multiple interactions promoted by ILs are critically discussed concerning the improved selectivity towards target molecules. Overall, the versatility of supported ILs is here considered a critical point to their exploitation as alternatives to the more conventional liquid chromatographic matrices used in bioseparation processes.

## 1. Introduction

Liquid chromatography is one of the most widely used methods in the field of biotechnology, both at the analytical and preparative levels. High-performance liquid chromatography (HPLC) consists in a chromatographic approach to separate, identify, and quantify specific components of a given mixture, for example, for the identification of constituents of a biological sample or separation of chemical compounds [1]. This technique is the method of choice for analytical procedures, and well-established operation modes, types, and stationary phases are recognized [2]. On the other hand, preparative chromatography consists of the effective purification of high-value products from complex mixtures [3]. Both types of chromatographic approaches rely on the distribution of a target molecule between the mobile and stationary phases. The performance of the preparative chromatography highly depends on the selection of efficient stationary phases with great selectivity towards the molecule of interest [3,4,5]. Initially, supports were designed for the separation of small molecules; however, with the increased demand for protein purification, novel matrices with enhanced robustness and selectivity were developed [6]. More recently, with the advances of nucleic acid-based therapeutics, new challenges are imposed to the purification strategies. Thus, novel adaptations to the chromatographic supports are required, and specific interactions are mandatory for developing effective chromatographic methods for the purification of these high-value biomolecules [7]. The techniques currently available to isolate and purify nucleic acids still present several limitations, enhancing the necessity of establishing new methods able to improve biomolecules quality to fulfill the requirements of the regulatory agencies. Amongst these purification processes, chromatography still remains the technique of choice due to its enhanced performance to achieve improved selectivity towards the target biomolecule [8]. Conventional purification protocols based on precipitation with salts, temperature, pH, and high molecular weight polymers have been replaced by highly selective and revolutionary strategies, such as affinity chromatography [9,10,11]; multi-modal chromatography occupies an important place in the bioseparation field, once it adds a new dimension to conventional chromatography procedures, such as ion-exchange, hydrophobic interaction, reversed-phase, or affinity [12,13]. Usually, multi-modal chromatographic matrices present multiple functional groups that cooperate in binding and elution steps. These groups offer the possibility of various interactions to occur, such as hydrophobic, aromatic, electrostatic, and hydrogen-bonding interactions. Initially, multi-modal matrices were composed of aliphatic hydrophobic groups and were mainly used for the extraction and purification of small organic compounds. These were not useful for biomolecules once they became strongly bound, and the elution required the application of organic solvents. Lately, new versions of multi-modal matrices composed of aromatic and charged groups have appeared in which interactions with the target product are not so strong and can be disfavored using mild conditions such as increased/decreased salt concentrations or changes in pH [14].

Amongst the possible ligands in chromatographic matrices, in recent years, ionic liquids (ILs) have been proposed to functionalize stationary phases, giving rise to supported ionic liquids (SILs) [15,16]. ILs are organic salts with a wide structural diversity, which can display a multi-modal behavior because they present positive/negative charged groups and can be tailored by the introduction of several functional groups and alkyl moieties of different lengths [15,16].

In this review, we emphasize the applications of ILs as ligands in liquid chromatography by presenting the different types of immobilization strategies on typical chromatographic supports, such as silica and polymers, while envisioning the growing potential of SILs as an alternative to the more conventional matrices already used in bioseparation processes, particularly relevant in the purification of biopharmaceuticals.

## 2. Biomolecules Purification by Preparative Chromatography

Given the increased applications of biomolecules such as proteins and nucleic acids as novel therapeutics, relevant efforts have been focused on improving the downstream processing, which usually accounts for 50% to 80% of the total production costs [17], as described above. In this sense, the separation and purification of biomolecules constitutes a major limitation of modern biotechnology processes [18]. In order to achieve not only high purity levels but also high yields of such biomolecules, it is mandatory to consider three essential factors: the type of ligand used, to which matrix it is attached, and the type of chromatography applied [19].

An ideal matrix must be of low cost, rigid, and highly porous and must allow high ligand substitution, which cannot interact with the sample [20]. Commonly used matrices correspond to agarose, cellulose, dextran, silica, and polyacrylamide, among others [21]. There are a wide variety of chromatographic supports used for the purification of different bioproducts, initially exploited in the purification of proteins and then used by pharmaceutical industries since the 1950s [7]. There are also many types of chromatography that explore different interactions between the target molecule and the ligand. Among them are ion-exchange chromatography, hydrophobic chromatography, multi-modal chromatography, and affinity chromatography [11]. Affinity chromatography is considered the election method for downstream processing, particularly in the capture of various types of biopharmaceuticals, due to its high selectivity [10].

Table 1 summarizes some examples of chromatographic matrices with different types of ligands that have been developed for the purification of biomolecules. In ion-exchange chromatography, there are plenty of commercial supports available. For example, Bo et al. [22] tested anion-exchange chromatography for adenoviral vectors purification. They tested four supports, namely Q Sepharose XL, Fractogel TMAE, Fractogel DMAE, and Fractogel DEAE. The resin Fractogel TMAE showed a great binding performance and real potential to purify adenovectors comparatively to the other three tested resins that presented lower dynamic binding capacity. The purification of supercoiled plasmid DNA (sc pDNA) was described by Silvia-Santos et al. [23], who tested a cationic multi-modal support, the Capto^TM^ adhere. The authors isolated the sc pDNA isoform from other impurities, such as RNA and open circular pDNA isoform, with a recovery yield of over 90%. In a different study, Hirsch et al. [24] evaluated a multi-modal chitosan-based chromatographic support for isolating lactoferrin directly from cheese whey without a previous clarification treatment. With sulfanilic acid-modified chitosan mini-spheres, the authors could capture 68% of lactoferrin, with a purity degree of 70%. Diogo et al. [25] studied the capacity of the support Sepharose CL-6B treated with 1,4-butanediol diglycidyl ether to separate nucleic acids by exploring hydrophobic interactions. It was observed that, when a nucleic-acid-containing mixture obtained from *E. coli* cells was loaded onto the column, it was possible to separate single-stranded from double-stranded nucleic acids. RNA and gDNA were more retained in the column due to interactions occurring with the hydrophobic exposed bases of these biomolecules, while pDNA was eluted in the beginning since nitrogenous bases were less exposed. In a recent study, a novel minicircle DNA (mcDNA) purification method was established by Almeida et al. [26]. It was possible to successfully isolate mcDNA from RNA, parental plasmid, and miniplasmid by size exclusion chromatography with a Sephacryl S-1000 SF matrix, achieving 66.7% of mcDNA recovery with 98.1% of purity. The same group of authors [27] proposed a mcDNA purification strategy with two modified monolithic supports. Lysine and cadaverine modified monoliths were tested for the isolation of mcDNA from other contaminants, such as other DNA isoforms and RNA. Cadaverine showed a better selectivity than the lysine modified support, enabling the successful mcDNA purification with 78.6% of recovery yield and 98.4% of purity. A different approach from Pereira et al. [28] was based on the development of an affinity chromatographic procedure for the purification of the micro-RNA-29 precursor (pre-miRNA-29). The L-arginine-Sepharose 4B chromatographic support was tested in this work, being able to successfully purify the pre-miRNA from other small RNA species, achieving a purity degree of 98%. Additionally, for the purification of sc pDNA, Azevedo et al. [29] tested three different chromatographic supports, histidine-agarose, arginine-macroporous, and histidine-monolith. The authors verified that, with arginine-macroporous, it was possible to reach a purity degree of 92%, but the recovery yield did not surpass 43%. However, this purified sc pDNA presented higher bioactivity levels compared to the pDNA purified with the other two supports. This might be because the main interactions established with the arginine ligand are of ionic character, while histidine mainly promotes hydrophobic interactions. Therefore, the mild conditions used for the elution with the arginine-based support contribute to an higher bioactivity, comparatively to the conditions used with histidine-based supports, in which high ammonium sulfate concentration has a significant effect on pDNA stability [29].

As mentioned above, despite the chromatographic supports already used, there are still several obstacles that need to be surpassed. There is still the need for the development of novel chromatographic ligands with better performance and characteristics, such as efficiency, reproducibility, selectivity, and being easy to obtain in a cost-effective manner. With these aims in mind, ionic liquids (ILs) could represent a valid option for the development of highly selective and more efficient chromatographic ligands for the purification of biomolecules and biopharmaceuticals.

## 3. Ionic Liquids

In the past decades, ILs have attracted the attention of many researchers due to their versatility and possible application in several areas, particularly emphasizing the potential to develop greener and sustainable processes [30,31]. The first IL was discovered by Paul Walden in 1914 when searching for molten salts that were liquid at temperatures at which his equipment could be used without special adaptations. Walden’s interest in these molten salts was the relation of their molecular size and their conductivity; however, the potential of this breakthrough went unnoticed for a long time. Almost 40 years later, the potential benefits of the lower melting points of molten salts was deeply recognized [32]. Today, ILs present a wide range of applications in various fields, such as organic, inorganic, physical, and biological chemistry [31,33].

ILs are organic salts that, in contrast with common electrolytes, display a wide range of possible interacions [34] and lower melting temperatures and thus can be liquid at room temperature. They are constituted by a large and nonsymmetrical organic cation such as imidazolium, pyrrolidinium, pyridinium, tetraalkylammonium, or tetraalkylphosphonium and numerous different inorganic or organic anions, such as chloride, bromide, acetate, or bistriflamide, among many other anions [35]. Since there are numerous possible cation and anion combinations, allowing the design of task-specific ILs, ILs are recognized as “Designer Solvents” [36,37]. Due to their ionic character, most ILs (if properly designed) present some outstanding features, including low volatility, non-flammability, variable viscosity and ionic conductivity, wide electrochemical potential window, high solvation ability, and excellent chemical, thermal, and electrochemical stability [36,37,38]. The first two characteristics contributed to the classification of ILs as “Green Solvents”, and, as a result, these compounds have been viewed as good alternatives to replace volatile organic solvents (VOSs) recurrently used in a wide range of processes. Ideally, this replacement would eliminate the loss of these solvents to the atmosphere and consequently reduce the harmful effects to the environment and human resources, making it possible to develop “greener” processes [37,39]. Nevertheless, other characteristics of ILs need to be taken into account before such claims can be made, such as their eco/citotoxicity and biodegradability. ILs are also usually recognized by their excellent solvation capacity for a wide range of compounds, as well as good stabilizing media for proteins, nucleic acids, and other bioproducts [36].

Besides being used as solvents, most of the time in their neat form or in aqueous solutions, ILs can be used in chromatography as ligands of the stationary phase, which is the main focus of this review paper [16]. With this, it is possible to combine the most powerful chromatography technique for biomolecules purification with the outstanding characteristics of ILs. Nevertheless, it should be noted that, once bound to a solid support, the cation/anion pair no longer constitutes a true IL. However, one of the most interesting properties of classic ILs, that is, their tunability, is maintained even when attached to chromatographic supports. The morphology of immobilized ILs varies, but the characteristics that depend on the structure of cation or anion can be preserved, ensuring the possibility to promote multiple interactions, such as hydrophobic, electrostatic, dipole–dipole, π–π, and hydrogen bonding [40]. Supported-ionic-liquids (SILs) maintain the valuable features of ILs with the addition of being supported, thus avoiding the use of large amounts of ILs, which can be costly and further prevent some toxicological concerns [41].

### 3.1. Supported Ionic Liquids (SILs) in Analytical Methods

ILs have been applied in several techniques, including extraction, chromatography, and spectroscopy. The growing interest of ILs in analytical chemistry is testified to by the increased number of publications that appeared during the last decade [42,43,44]. It should be mentioned that an impressive amount of works aimed to develop new stationary phases for HPLC, in order to improve column efficiency, permeability, and stability [45]. Reports concerning the applications of SILs have mainly focused on silica functionalization for gas chromatography [46]. Additionally, applications of ILs as stationary phases for hydrophilic interaction chromatography (HILIC) separations started to emerge [47]. In this review, we will focus on the use of ILs in solid supports for biomolecules separation and purification by means of liquid chromatography.

In the last years, advances towards the covalent immobilization of ILs onto silica materials and in the attachment of ILs onto polymers have been faced [46]. ILs can be immobilized in different ways, but always requiring interactions between the ionic liquid or its components (anions or cations) and the support material or functional group (or active species) [48].

IL-based supports received relevant attention due to their excellent properties and potential application in many fields of analytical chemistry. IL-modified polymers constitute better alternatives to the funtional porous polymers, such as sepharose and other commonly used copolymers, that have been used as stationary phase in HPLC separation once, besides achieving great selectivity, they also offer increased column efficiency [35]. More detailed information about stationary phases already used in HPLC can be found in the works of Vidal et al. [46] and Pino and Afonso [45].

### 3.2. ILs as Ligands of Chromatographic Supports

**Figure 1:**
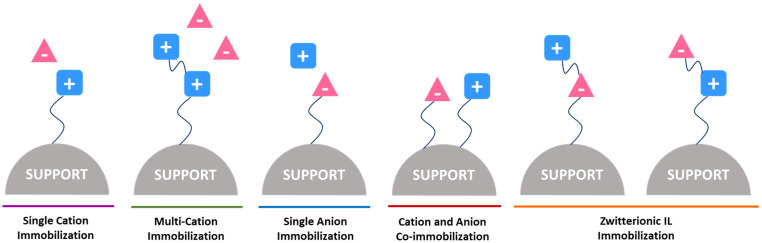
Illustration of distinct methods of IL immobilization onto a chromatographic support. From left to right: In the purple line is the cation immobilized on the support while the anion is free; in the green line is represented a multi-cation immobilization, with multi-anion as counter-ions; in the blue line is the anion immobilized on the support, while the cation is free; in the red line is represented the cation and the anion co-immobilized, where both are covalently bound to the matrix; In the orange line is represented zwitterionic IL immobilization, where the cation and anion are linked through a covalent bond.

Usually, when considering liquid chromatography, ILs are immobilized on the surface of solid supports by covalent bonding of their cations or anions [16], which is highly relevant to avoid the IL leaching. There are six ways to perform the IL immobilization onto the support, as shown in Figure 1.

In Figure 1, represented by the purple line, is the case where the IL cation is covalently attached to the solid support while the anion acts as a free counterion. This case is relatively easy to prepare and has the advantage that free anions can be easily replaced, making a simple regeneration processes, or even exchanged, enabling slight modifications by changing the anion [16]. Spherical porous silica is often used as a stationary phase matrix for HPLC, in which an imidazolium ring (the most used IL for covalent modification) is immobilized with a spacer arm. This type of stationary phase has already proven to be efficient in the separation of alkaloids, inorganic anions and cations [49], xylose, and glucose [50]. Additionally, the imidazolium-based support can promote different types of interactions, demonstrating hydrophobic and ionic properties, and, hence, enabling multi-modal separations. Usually, the cation is anchored on the silica by a small spacer arm, and different lengths of these spacers may influence the selectivity of the support by alteration of hydrophilicity [17,41,51]. Moreover, in a study performed by Neves et al. [52], where a macroporous support was functionalized with 1-methyl-3-propylimidazolium chloride, the establishment of different types of interactions between the ILs and biomolecules, such as gDNA and RNA, was also proven, enabling the effective separation of these two species. Several aspects point to the potential of ILs as truly multi-modal ligands, considering their ability to interact with analytes through different mechanisms, including hydrophobic, electrostatic, hydrogen bonding, π–π, and dipole–dipole interactions, due to their unique structure that comprises hydrophobic, hydrophilic and ionic moieties.

The green line in Figure 1 also represents an IL immobilization with multi-cation moieties. In a study from Qiao et al. [52], it was shown that stationary phases with immobilized dicationic ILs presented effective retention and good selectivity for typical hydrophilic compounds under the HILIC mode, as well as an improved column efficiency. On the other hand, anions are rarely immobilized onto the supports (Figure 1, blue line), since a particular study of Qiu et al. [53] demonstrated that, in this case, the counterions are easily exchanged by the ionic species present on the mobile phases during usage. This effect greatly compromises the stability of the IL-based support and restrains the reproducibility of separations due to the possible exchange of the counterions affecting the interactions established.

The red line in Figure 1 shows the co-immobilization of the cation and the anion onto the solid support. This strategy can improve the stability of these ligands during the use of different types of buffers in the mobile phase, thus influencing the selectivity by the distribution of polar groups [40,54]. Finally, in the orange line of Figure 1 are given the examples of zwitterionic compounds, which can avoid the previous issues, once the cation and anion are covalently bond to each other, varying between which one is immobilized onto the solid support. Qiao et al. [55] developed a zwitterionic stationary phase with a positively charged imidazole ring and a negatively charged sulfonate group, which exhibited good selectivity and favorable retention for a wide range of polar solutes, including nucleosides and nucleic acids.

Thus, ILs allow a wide variety of interactions since they present both a positive and negative charge, which enables many electrostatic interactions according to the buffer pH and the biomolecule of interest. Furthermore, they also present functional groups, such as an imidazole ring, amine, and carboxyl groups that promote several types of interactions (hydrogen bonds and van der Waals, among others). They even can have alkyl chains (with various lengths) that strongly favour hydrophobic interactions with the different molecules of interest. All of this justifies ILs’ applicability as promising ligands in chromatography once they can act as multimodal ligands [14,36].

## 4. Silica Supported Ionic Liquids—SSILs

Due to its versatility and physical characteristics, silica is one of the most-used stationary phases in chromatography. A great advantage of using silica is that a wide range of selectivity can be achieved since it can be easily modified or functionalized by the immobilization of different compounds to siliceous particles. In contrast, one important disadvantage of silica-based materials occurs upon the analysis and separation of basic compounds. In this case, strong interactions can be established between these compounds and the free residual silanol groups at the surface of these supports [45], limiting the effective separation. This shows how crucial it is to perform the appropriate modification of the surface of the stationary phase. Hence, it is highly important to know all the possible chemical immobilization routes and identify the best options for the linkage of a certain ligand.

### 4.1. Single Cation Immobilization

#### 4.1.1. Immobilization via Halogenated Silane Groups

**Figure 2:**
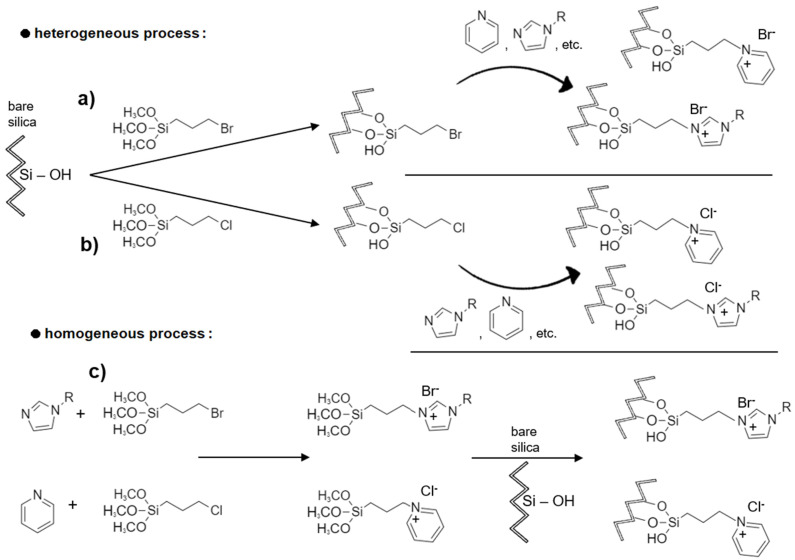
General processes for the single cation immobilization routes of silica supported-ILs via halogenated silane groups. Chemical routes for the heterogeneous process, using the 3-bromopropyltrimethoxysilane (**a**) or 3-chloropropyltrimethoxysilane (**b**), and the homogeneous process (**c**). (Chemical structures were design at ChemDraw^®^ software).

One of the first works reporting the use of ILs in the modification of silica surface dates from 1985, where Gushikema and Moreira [46] used silica gel functionalized with 3(1-imidazolyl)propyl groups to adsorb compounds. In fact, the imidazole group covalently attached to the silica surface is the leading example of the use of SILs in SPE [46]. This functional group can be immobilized onto the silica surface by different means, such as grafting 3-bromopropyltrimethoxysilane to a silica substrate (Figure 2a). The subsequent modification of the linker and endcap-modified silica with an ionic liquid precursor compound, usually the imidazole (others might include alkylimidazole, pyridine), leads to the formation of the desired SIL [45]. Another immobilization method also uses activated silica, but the modification occurs with another silane-coupling agent, the 3-chloropropyltrimethoxysilane (Figure 2b). Both modification approaches are obtained by the heterogeneous process [45]. The requisite is that the silane coupling agent possesses a halogen at the terminal position to quaternize the imidazole, pyridine ring, or other tertiary amines.

One of the first works showing the application of N-Methylimidazolium chloride in a silica stationary phase for high-performance liquid chromatography was shown by Qiu et al. [56]. In this work, a N-methylimidazolium functionalized silica (the SilprMimCl) was synthesized by the methodology described above (Figure 2b), in which activated silica was suspended in toluene, and then an excess of 3-chloropropyltrimethoxysilane was added, followed by triethylamine addition, to act as a catalyst. The suspension was mechanically stirred and refluxed for 48 h, and the chloropropyl silica (SilprCl) was obtained. Afterwards, the chemically bonded chloropropyl group on the silica surface reacted with an excess of N-methylimidazole. The mixture was again refluxed with stirring for 48 h. After proper washing and drying, the SilprMimCl was obtained and used for the separation of a mixture of common inorganic anions by anion-exchange chromatography. The authors concluded that the SilprMimCl acts an anion-exchange phase, but it has multi-modal retention properties since it also allows the establishment of reverse phase interactions. This finding suggested that this SIL could be interesting for application in the separation of some biological samples, such as amino acids and proteins, among others [56].

The same functionalization method (Figure 2b) can also use pyridine as the source of the ionic liquid cation instead of imidazole. Moreover, Auler et al. [57] have reported propylpyridinium chloride groups on the silica surface for a novel stationary phase to separate organic compounds and inorganic anions by anion-exchange chromatography. This was performed by a two-step modification process, in which the first part is common to others, which means that silica particles were silanized with 3-chloropropyltrimethoxysilane to yield SilprCl. Then, in the second part, the modified silica was reacted with pyridine to produce positively charged propylpyridinium groups on the silica surface, plus the chloride anion (SilprPy). This novel SIL matrix performed HPLC separations of common inorganic anions and some non-polar and polar compounds. The results showed good efficiency and resolution parameters, indicating that this new chromatographic support is promising for the analysis of these compounds in environmental samples [57].

A different approach to obtain the same kind of functionalized SILs is called the homogeneous process (Figure 2c). In this process, the IL is synthesized by reacting either the 3-chloropropyltrimethoxysilane or 3-bromopropyl-trimethoxysilane with an imidazole or pyridine compound, and then, the resulting IL is easily bound to the silica.

Despite the differences described in the three described functionalization mechanisms (Figure 2), all of these SILs were prepared by a nucleophilic substitution reaction between the imidazole/pyridine groups and the halogenated alkane molecules (R—Cl/Br). All three correspond to an immobilization of ILs via the cation (Figure 1, purple line). This means that the number of different SILs that can be produced by these mechanisms is quite limited because few nucleophilic groups are available with the right conditions (tertiary amines). On the other hand, the commercially available halogenated alkanes with other functional groups are also very scarce. Nevertheless, additional SILs can be prepared by metathesis reaction to exchange the anion. Almeida et al. [58] described several similar SILs prepared from the SilprMImCl differing on the anions. To accomplish the anion exchange, the SilprMImCl was mixed with an excess of the anion source in an aqueous solution and was left under moderate stirring and temperature for 24 h [58]. Bi et al. [50] also described similar methodologies where NaBF_4_ was added to SilprImCl in dichloromethane and stirred for 24 h to obtain SilprImBF_4_. The substitution procedure can be accomplished in batch mode by soaking the IL-immobilized silica or on-column by filling it with the IL-immobilized silica and adding a highly concentrated solution of the desired anion. However, this metathesis reaction presents as a downside the fact that new anions tend to run off gradually or be replaced by other undesired anions [40].

Recently, Liu et al. [59] proposed a greener “solvent-free” method to synthesize an N-methylimidazolium-grafted silica stationary phase. The silica and 3-chloropropyltrimethoxysilane were added to the N-methylimidazole in excess, without adding any extra organic solvent. This “solvent-free” achievement is possible due to the addition of the functional group in a liquid state and at large excess.

The application of ionic liquids in liquid chromatography gained a particular interest in the last decade, particularly in the exploitation of mixed-mode interactions. In 2010, a novel amino-imidazolium stationary phase based on silica was synthesized and characterized by Bi and Row [60]. The authors used this stationary phase to separate some compounds that could not be easily separated by HPLC using the traditional C18 column. SilprImCl was synthesized following the method described in Figure 2b and mixed with ethanol and 3-bromopropylamine hydrobromide. The suspension was then refluxed with stirring for 24 h. After washing and drying, the amino-imidazolium bromide silica (SilprImNBr) was successfully obtained. Unlike the traditional C18 column, the SilprImNBr stationary phase enabled the separation of different aromatic organic compounds and alkaloids, which can be attributed to the multiple interactions promoted by the SilprImNBr [60]. Later, Yang et al. [61] investigated the differences between 1-methylimidazolium chloride (Sil-1-MImCl) and 2-methylimidazolium chloride (Sil-2-MImCl) functionalized silicas for mixed-mode chromatography application. The preparation of Sil-2-MImCl and Sil-1-MImCl was achieved by the mechanism represented in Figure 2b, where the functional monomer was the 2-methylimidazole or 1-methylimidazole, respectively. Using these SILs, the authors [61] reported the separation of nucleosides, nucleobases, water-soluble vitamins, sulfonamides, and saccharides by hydrophilic chromatography (HILIC), and the separation of inorganic anions by anion exchange chromatography (AEX). The obtained SIL matrices demonstrated good chromatographic performance for both HILIC and AEX modes [61].

Information on the SILs immobilized via halogenated silane groups, structure, analyte type, and corresponding application technique is provided in Table 2. Abbreviations for SILs are given as indicated in the original paper, except for some examples where others were employed to avoid conflicting terminology. As seen in most examples presented in Table 2, 1-alkylimidazoles with short aliphatic chains, including methyl, ethyl, and butyl, and with the aromatic benzyl group, are among the most described. This is due to their commercial availability. Other imidazoles may require previous functionalization, for instance, the synthesis technique for the preparation of multiple-substituted imidazole derivatives can be performed by the so-called Radziszewski reaction, which was initially the industrial synthesis strategy to produce imidazole [40]. This generates imidazoles with one or more substituents by cyclocondensation of ring fragments, typically by combining glyoxal, ammonia, an aldehyde, and a primary amine.

#### 4.1.2. Functionalization via Modified Stöber Method Incorporating Functional Alcohols

An alternative route to synthesize functionalized silica particles incorporating alcohol compounds through the Stöber process has been described by Lee et al. [72]. The Stöber method is one of the most common methods to synthesize nanoparticles due to its simplicity and reliability. This method involves the hydrolysis and condensation of silicon alkoxide precursors, such as tetraethyl orthosilicate (TEOS) in an ethanol solution, using water and a base (e.g., ammonium hydroxide) as catalysts. In particular, the authors [72] chosen cholinium chloride (ChCl) as a functional alcohol to create quaternary ammonium functionalized SILs. Usually, the formation of quaternary ammonium functionality on the silica surface comprises a multi-step process involving silanes and other compounds [73]. However, Lee et al. [72] have reported this distinct one-step process that can be easily accomplished by incorporating cholinium chloride into the Stöber synthesis. In detail, the hydroxyl group of cholinium chloride and silanol groups created during hydrolysis of tetraethyl orthosilicate (TEOS) can react by condensation, forming silyl ether (Si-O-C) bonds and exposing a positively charged quaternary ammonium group at the surface of the particles [72]. The results showed an increase of the zeta potential (from 3 to 10) of silica particles incorporating cholinium chloride. Thus, it is clear that the incorporation of ChCl in the Stöber synthesis could significantly alter the surface of the silica particles to a positively charged functionality. However, it should be pointed out that the Stöber process is an example of a sol–gel process, i.e., one-pot synthesis (co-condensation method), with the organic groups being grafted into the inner and outer pore walls during their formation. Hence, the organic limit for this modification is 40 mol % to avoid pore disorganization [74]. On the contrary, if the functionalization occurs just after the pore formation (immobilization of silica gel particles), the modifier is attached only to the outer pore walls, and the organic limit can be much higher. Thus, other means for the preparation of quaternary ammonium(Ch)-functionalized silica would be preferred (example shown below [73]).

#### 4.1.3. Immobilization via Thiol-Containing Silanes

**Figure 3:**
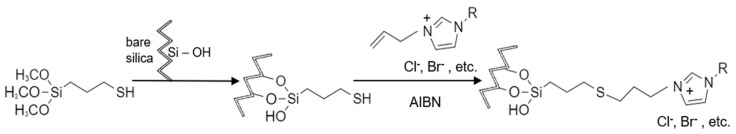
Scheme of the process for the cation immobilization of silica supported-ILs via thiol-containing silanes (through “thiol-ene” click reaction). (Chemical structures were designed using the ChemDraw^®^ software).

Another approach starts by linking the ILs to the surface of 3-mercaptopropyl modified silica through the so-called “thiol-ene” click reaction [47]. This is performed by modifying the activated silica with another silane-coupling agent, the 3-mercaptopropyltrimethoxysilane (MPTMS). Then, the MPTMS-modified silica reacts with a specific IL, which must contain a 1-allyl as substituent of the imidazolium cation, in the presence of azodiisobutyronitrile (AIBN) [40] as the initiator, via the radical chain-transfer addition reaction [45] (Figure 3).

Qiao et al. [69] developed an imidazolium-embedded C8 stationary phase with L-lactate as the anion for mixed-mode chromatography. This was easily prepared by the two steps presented in Figure 3. First, MPTMS-modified silica was prepared, and then the 1-allyl-immidazolium IL (1-vinyl-3-octylimidazolium L-lactate) was bonded to silica via surface radical chain transfer reaction. This novel imidazolium-embedded C8 stationary phase indicated both RPLC and HILIC retention mechanisms, meaning that both hydrophobic and hydrophilic compounds could be simultaneously separated through a single column. The new support was further used to successfully separate a mixture composed of both hydrophobic compounds (naphthylamine, nitroaniline, phenanthrene, terphenyl, and triphenylene) and hydrophilic compounds (cytidine and uracil). When the same sample was separated by a commercial C8 column, the peaks overlapped together. In a different assay, this imidazolium-embedded C8 stationary phase with L-lactate as anion, was used for the separation of a highly polar component from a complex mixture of *Trichoderma* sp. metabolites [69] by mixed-mode RPLC/HILIC. Overall, the novel imidazolium-embedded C8 stationary phase was demonstrated to be promising for the separation of complex samples with different physicochemical properties.

More recently, Qiao et al. [70] reported the preparation of an amide-functionalized imidazolium IL-bonded silica stationary phase by the same methodology (Figure 3). A prepared 1-vinyl-3-amide-substituted imidazolium IL was grafted onto the surface of the 3-mercaptopropyl modified silica through “thiol-ene” click chemistry [70]. Chromatographic performance and separation selectivity of this silica surface-bonded amide-functionalized imidazolium IL were evaluated and compared with a commercial Tosoh amide column (calculated theoretical plate number was 69,000/m (k 3.68) for the Tosoh amide column and 70,000/m (k 3.54) for the amide IL column) [70]. The novel matrix exhibited superior separation performance towards typical hydrophilic solutes from complex samples, such as flavonoids mixture, soybean flavonoids, and human urine. The results indicate that the combination of the amide group and imidazolium IL moiety bring some advantages in selectivity as a novel stationary phase, as this novel amide IL column possessed an anion-exchange/HILIC mixed-mode retention mechanism [70].

Besides imidazolium-based IL, glucaminium-based ILs were also exploited. In the same group, Qiao et al. [52] prepared a glucaminium-based IL, named N,N-diallyl-N-methyl-d-glucaminium bromide, through nucleophilic substitution between N-methyl-d-glucamine and allylbromide. Afterwards, the resulting glucaminium-based IL was bonded onto the silica surface by “thiol-ene” click chemistry [52]. Then, the retention behavior was evaluated using different solutes, with the results revealing that the developed surface-confined glucaminium-based IL stationary phase exhibited a hydrophilic interaction/anion-exchange mixed-mode retention mechanism. This result can be explained by the fact that the obtained glucaminium-based IL column possessed a hydrophilic glucose structure and positively charged quaternary ammonium group immobilized onto silica, which provided the possibility to interact with solutes by either hydrophilic interaction or anion-exchange. Furthermore, mixtures of nucleotides and flavonoids were separated on this glucaminium-based column under hydrophilic interaction and hydrophilic interaction/anion-exchange mixed-mode chromatography. It was concluded that the multi-modal retention capabilities of this glucaminium column could offer a wide range of retention and flexible selectivity toward several polar and hydrophilic mixtures of compounds.

#### 4.1.4. Immobilization via Amine- or Hydroxyl-Containing Silanes

**Figure 4:**
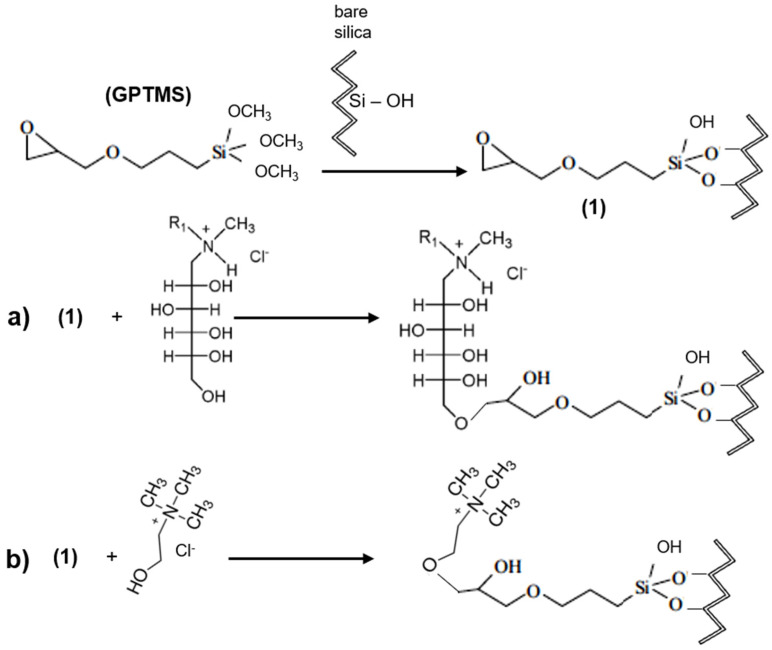
Schematic representation for the preparation of novel silica-based stationary phases functionalized with bio-based ILs, such as glucaminium-based ILs (**a**), cholinium-based ILs (**b**), via the epoxy group of the glycidyloxypropyltrimethoxysilane through “click” chemistry reactions. (Chemical structures were designed using the ChemDraw^®^ software).

Besides the aforementioned methods, there are other effective reaction schemes for preparing SILs, which do not encompass the use of CPTMS and MPTMS, the more dominant coupling agents. Limiting the preparation of SILs to only two functional silane groups, i.e., haloalkyl and mercapto, was very insufficient for the development of a vast range of multi-functional supported ILs. Thus, other silane coupling agents might include aminopropyltrimethoxysilane (APTMS), glycidyloxypropyltrimethoxysilane (GPTMS), and n-(2-aminoethyl)-3-APTMS (EAPTMS). These groups are highly reactive and most of the reactions occur through “click” chemistry. One example of this is the use of the coupling agents GPTMS, which contain an extremely versatile epoxy group (oxirane), enabling further functionalization by a large range of nucleophiles, and electrophiles under mild conditions [40]. Another feature of click chemistry is that these epoxy groups can undergo ring-opening, under solvent-free conditions. In the case of imidazole, for instance, it would form imidazolyl alcohols. Hence, silica surfaces covalently coated with this silane coupling agent (GPTMS) can be easily used to conjugate thiol-, amine-, or hydroxyl-containing ligands, only varying the pH of the reaction [75]. Figure 4 schematically presents the reaction for hypothetical preparation of novel silica-based stationary phases functionalized with bio-based ILs, such as glucaminium-based ILs (Figure 4a), cholinium-based ILs (Figure 4b), or other ILs that include thiol-, amine-, or hydroxyl-functional groups.

Zhang et al. [40] suggested the use of GPTMS to react with imidazole to generate a new silane coupling agent, where further quaternization by an haloalkane on the 3-position of imidazole could produce new SILs. Zhou et al. [76] prepared a SIL for chiral separation through binding of 1,2-dimethylimidazole or 1-amino-1,2,3-triazole substituted 6-tosyl-β-cyclodextrin chemically onto silica. After preparing the imidazolium or triazolium-β-cyclodextrin derivatives, GPTMS reacted with the hydroxyl group of the β-cyclodextrin derivatives. Then, silica gel was added to form the final chiral SILs. By using these novel chiral SILs, outstanding enantio-separations were achieved for chiral separation of aromatic alcohol derivatives and racemic drugs. Moreover, these results showed that both the imidazolium cation and its counter anion could contribute to the ultimate chiral resolution [76]. On the other hand, APTMS contains a short organic 3-aminopropyl group, which terminates in a primary amine. Primary amines react with carbonyl compounds to yield imines, or with acid chlorides to form amides. Additionally, primary amines can react with alkyl halides to form an ammonium salt (or IL) and via epoxy leading to a ring-opening. Tian and Row [77] used APTMS to react with 3-chloropropionyl chloride to bear the corresponding amide-modified silane, which then reacted with imidazole to synthesize the imidazolium-functionalized silane. The selectivity of the obtained ionic-liquid-modified silica was successfully used in the solid-phase extraction (SPE) to isolate tanshinones from *Salvia miltiorrhiza* Bunge [46]. This novel SIL exhibited a higher selectivity than traditional silica and C18 cartridges [77].

In its turn, the EAPTMS is a silane coupling agent very similar to the APTMS with an extra amine in the middle. This reagent enables the formation of an IL-functionalized silane with at least two secondary amines that can act as hydrogen bond donors and the oxygen of the amide as a potential hydrogen bond acceptor when interacting with biocompounds. Thus, the corresponding SIL becomes an excellent choice for a HILIC or multi-modal chromatography ligand.

Other substituted silanes may be available, but most of the chemistry principles behind the functionalization reactions have already been described. Carpenter et al. [73] used a similar silane coupling agent, namely the N-(6-aminohexyl)aminopropyltrimethoxysilane (6 carbons in between the amines), for the synthesis of quaternary ammonium-functionalized silica nanoparticles. The synthesis strategy first involved the reaction of epichlorohydrin (chlorinated epoxy compound) with a series of dimethylalkylamines to obtain glycidyltrialkylammonium chlorides, with diverse alkyl chain lengths, and then its reaction with the surface of amine-containing silica nanoparticles occurred via a ring-opening reaction [73].

### 4.2. Multi-Cation Immobilization

Until recently, SILs’ stationary phases for HPLC were mostly based on single-cation ILs, but Qiao et al. [52] introduced the multi-cationic ILs (Figure 1, green line) in the preparation of different IL-based stationary phases for liquid chromatography. Both dicationic and tricationic SILs were prepared by these authors [52,78]. Typically, dicationic SILs (Figure 1, green line) contain two cations linked by a spacer arm and two counter anions, while tricationic SILs are composed of three positively charged moieties anchored to a central core and three coordinating anions [16]. When multi-cationic ILs are immobilized to the silica surface, they have the potential to enhance the ionic nature and hydrophilicity of the support, favoring the retention of typical biological compounds under HILIC or mixed-mode manner. Geminal dicationic ILs 1,4-bis(3-allylimidazolium)butane or 1,8-bis(3-allylimidazolium)octane di-bromide (C4/8DiImBr) and the 1,4-bis(3-allylimidazolium)butane or 1,8-bis(3-allylimidazolium)octane di-bis(trifluoromethanesulphonyl)imide (C4/C8DiImNTf2) were synthesized, and these ILs were then bonded to stationary phases by “thiol-ene” click chemistry, using the MPTMS to modify the silica surface. Additionally, the authors prepared monocationic ILs, namely the 1-vinyl-3-octylimidazolium and 1-allyl-3-butylimidazolium bis(trifluoromethanesulphonyl)imide (C4/C8ImNTf2), and added them to the silica particles in the same manner [52]. Afterwards, the dicationic SILs were compared with their monocationic analogues, verifying that the dicationic SILs displayed an effective retention and good selectivity for typical hydrophilic compounds (nucleosides and nucleic bases) under HILIC mode, presenting a column efficiency as high as 130,000 plates/m [52]. Later, the same authors reported the preparation of two tricationic SILs as new stationary phases for HPLC, through “thiol-ene” click chemistry [78]. The obtained tricationic SILs were evaluated both in RPLC and HILIC mode. These stationary phases have been shown to exhibit good selectivity to hydrophobic compounds, presenting an ideal column efficiency of 80,000 plates/m in the RPLC mode, with naphthalene as the solute. When compared with a commercial C8 column, the tricationic SILs have weaker hydrophobicity, but exhibit a remarkable selectivity to isomeric PAHs, due to multiple interactions (π–π and dipolar interactions). Regarding the HILIC mode, the retention and selectivity were evaluated through the separation of nucleosides, nucleic bases, and flavonoids. Typical HILIC retention behavior was demonstrated by investigating retention changes of hydrophilic solutes with aqueous mobile phases [78]. The results showed that these columns exhibited more efficient retention and better selectivity towards flavonoid standards when compared to reported standard HILIC columns, concluding that these tricationic SILs hold a great prospect for the analysis of more complex samples.

### 4.3. Single Anion Immobilization

All the presented functionalization mechanisms described so far correspond to immobilization of ILs via the cation; however, ILs can also be immobilized to the silica surface via the anion. Again, different approaches could be implemented, described in the following topics.

#### 4.3.1. Immobilization via Lewis Acidic Chloroaluminate Ionic Liquids

This synthetic route is usually described as the incipient “wetness method” or immersion method. For this, the IL is slowly added to the support material until the latter has lost the appearance of a dry powder. The excess of liquid can be then removed by means of Soxhlet extraction. It is important to notice that the support material is impregnated in a pre-designed functional ionic liquid that must contain the chloroaluminate group, for the covalent anchorage of the anion group to the support surface via chloroaluminate-based anion [79]. The immobilization of the IL to the support is due to the formation of covalent bonds between the aluminum atoms of the anion and the hydroxyl groups [79] of the silica particles surface. A possible drawback of this method is that the structure of the support may be destroyed when structured support materials, such as zeolites or silica supports of the MCM family, are used [79].

#### 4.3.2. Immobilization via Functional Silanes

Another possible route of synthesis for the preparation of SILs immobilized via the anion is to use the triflyl group, CF_3_SO_2_ (abbreviated Tf_2_O), and convert a functional silane (APTMS) into bis(trifluoromethanesulfonyl) imide-modified silane by a trifluoromethanesulfonylation step, followed by a lithiation reaction to obtain the SiO_2_-NTf-Li. Trifluoromethanesulfonic anhydride ((CF_3_SO_2_)_2_O), a strong electrophile, can easily react with the amine of APTMS. Then, lithium (Li^+^) in previously prepared SiO_2_-NTf-Li can be substituted through ion exchange by other more common cations of ILs, such as 1-butyl-3-methylimidazolium (BMIM^+^) and tetrabutylammonium (NBu4^+^) [80].

Some amino acid(AA)-based ILs, which are typically composed of imidazolium cations and amino acid anions, possess hydroxyl, amine, or thiol groups in the AA anion [81]. Hence, silica surfaces covalently coated with GPTMS can be easily used to conjugate some of these AA-based ILs via the anion, for instance the [Ser]^−^, serinate; [Thr]^−^, threonate; [Lys]^−^, lysinate; [Tau]^−^, taurinate; [Val]^−^, valinate; and [Cys]^−^, cysteinate.

### 4.4. Co-Immobilization of the Anions and Cations

As previously mentioned, the use of pure alkyl stationary phases (e.g., C18) still has some disadvantages, such as low compatibility with highly aqueous eluent and insufficient selectivity towards more polar solutes. Despite this, C18 and C8 are still amongst the most commonly used support phases in reverse-phase liquid chromatography (RPLC), in which a hydrophobic stationary phase and a polar (aqueous) mobile phase are used. Zhang et al. [67] were able to synthesize novel stationary phases based on modified alkyl imidazoliums to form polar-embedded phases. These polar-embedded phases have lower hydrophobicity compared to C18 phases and are notable for their stability in aqueous media, enhanced performance in separation of polar compounds, and higher selectivity. One of the new phases reported in this study was obtained via monomeric immobilization of 1-octadecylimidazole to 3-chloropropyltrimethoxysilane-modified silica (SilprImCl) to form a polar-embedded phase (SilprImC18Cl). Another support was prepared by the co-immobilization of two silane coupling agents (3-chloropropyltrichlorosilane and octadecyltrichlorosilane) to silica, followed by addition of methylimidazole to the SilprCl reaction sites to form a polar-spaced phase (SilprMImCl+SilC18). It was concluded that the co-immobilized SilprMImCl with C18 showed a higher selectivity factor, and it was also the phase that presented higher density of C18 ligands. However, the presence of an imidazolium core was important to enhance aromatic selectivity and also weaken the hydrophobicity character of the long alkyl chain [67]. Despite the novelty, introduced in this work with the co-imobilization of C18 and methylimidazole, only the latter had the character of SIL. More recently, the effective co-immobilization of two different ionic liquids on silica particles was described (Figure 1, red line) [40,54]. Wang et al. [66] compared the performance of silica modified with mono-immobilized and co-immobilized imidazolium ionic liquid for the removal and recovery of 2,4-dinitrophenol (2,4-DNP) from aqueous solutions. The functional monomer was the typical imidazole ring (Im) substituted with different groups, including N,N-dimethylaminopropyl (A), benzyl (Bz), dodecyl (C18), and naphthylmethyl (Naph). SilprImACl, SilprImBzCl, SilprImNaphCl, SilprImC18BzCl, and SilprImC18ACl were then synthesized by a combination of the several reaction mechanisms described in Figure 2. The experimental results revealed that the substituent groups such as N,N-dimethylaminopropyl, benzyl, and naphthylmethyl on the imidazole ring can significantly enhance the adsorption of 2,4-DNP via the π–π interaction and that the adsorption capacity of 2,4-DNP follows the order: SilprImNaphCl > SilprImACl > SilprImBzCl. Additionally, SilprImC18BzCl exhibits the largest adsorption capacity while SilprImC18ACl has the lowest among the five adsorbents. Thus, the combination of hydrophobicity and π–π interactions led to enhanced adsorption performance towards 2,4-DNP from aqueous media [66].

### 4.5. Cation or Anion Immobilization Using Zwitterionic ILs

Generally, imidazolium IL-based HPLC stationary phases display good retention behavior due to the potential to interact with solutes through multiple interactions, including hydrophobic, electrostatic, and hydrogen-bonding interactions, among others. However, regarding hydrophilic interactions, there are only a few reports on imidazolium ILs as HILIC stationary phases. Efforts have been made in order to prepare chromatographic supports with ligands, containing both positive and negative charges at a stoichiometric ratio (Figure 1, orange line), which can be more suitable for the separation of polar analytes. Thus, a new class of zwitterionic IL stationary phases for HILIC has emerged, showing excellent selectivity and efficient retention for various polar solutes [55]. These zwitterionic stationary phases can be synthesized by binding the zwitterionic compound to the surface of 3-mercaptopropyl modified silica particles by “thiol-ene” click chemistry. Qiao et al. [55] have shown the synthesis and application of a new imidazolium-based zwitterionic IL, where they used the zwitterionic 1-vinyl-3-(butyl-4-sulfonate)imidazolium, synthesized by a complete reaction between 1-vinylimidazole and 1,4-butane sultone without any by-products. Differently, Shen et al. [82] have prepared a cysteine-bonded stationary phase that belongs to zwitterionic stationary phases, also by “thiol-ene” click chemistry. However, in this case and unlike the typical zwitterionic stationary phases, the distribution of oppositely charged groups was paralleled to the surface of the silica gel due to the small conformation of the amino acid. For this functionalization, the vinyl group was present in the silane group that was used to functionalize the silica particles, and the thiol reactive group was the free side chain of this particular amino acid. In detail, the trichlorovinylsilane was used to functionalize silica particles, and the thiol group of cysteine was left to react via the “thiol-ene” click reaction in the presence of AIBN to obtain the resulting material “Click TE-Cys” [82].

## 5. Polymer-Supported Ionic Liquids—PSILs

Silica supports are very interesting for most of analytical chromatography methodologies, in which small molecules can be efficiently separated from biological samples. However, for the purification of larger biomolecules, some limitations can be found because of the limited pH stability. In addition to silica supports, polymers have been functionalized as well with ILs, mainly due to their high relevance in preparative chromatography, especially for the purification of added-value biomolecules, in the sense that they allow a wider operating pH range, important for cleaning regimes, whereas NaOH solutions are commonly used. As with silica, the properties of polymers to be used as SPE materials can be improved by immobilizing them with ILs [46]. Regarding the polymer supported ILs (PSILs), imidazole and pyridine groups have been the most used in the polymerization process [46]. The first work in this field was published in 2009 by Fontanals et al. [83], where N-methylimidazolium trifluoroacetate was part of the polymer. This novel IL-polymer was used as an SPE sorbent for the extraction of ten pharmaceutical products from water samples, which are emerging pollutants in the environmental field [83]. Dealing with other types of samples and application, Bi et al. [84] studied the applicability of an aminopropyl-imidazolium polymer as sorbent for the extraction of matrine and oxymatrine from *Sophora Flavescens Ait*. This PSIL exhibited higher selectivity than commercial C18 and amino-based sorbents. The authors [46] have concluded that the existence of an IL group protects the amino group against protonation; however, the role of the amino group on the extraction is not clear [46]. Probably, the presence of amino groups in these supports can be also valuable upon purification of more complex biomolecules, such as proteins and nucleic acids. A molecular imprinting technique was also applied to incorporate functional IL-groups in the porous structure of a PSIL with 9,10-phenanthrenequinone as the template [85]. Five IL-modified polymers with different functional groups were studied by Tian et al. [85]. The porous polymer was prepared firstly by co-polymerization of 4-(chloromethyl)styrene and divinylbenzene, and then five imidazoles with different functional groups (imidazole, methylimidazole, carboxyl-imidazole, amino-imidazole, and cyano-imidazole) were used as surface modifiers to form IL structures. From the five IL-polymers, the carboxyl-imidazolium exhibited the highest extraction efficiency and the best selectivity due to the strongest hydrogen-bonding interaction with the carboxyl group [46].

With a different chemical structure, an alkyl-pyridinium polymer material was prepared for the extraction of liquiritin and glycyrrhizin from Licorice [64]. In this work, the PSIL did not include the previously introduced imidazolium cation group but instead a pyridinium group. The IL-polymer was compared with a C18 sorbent, showing that the pyridinium-based PSIL has higher selectivity [46,64]. The reported result reveals that under the same washing and elution condition, C18 cannot separate the LQ and GA [64].

Although there are already some works reporting the use of PSILs in separation processes, most of these works are still focusing on small molecules or metals separation for analytical methods. Hence, there is a demand for creating novel chromatographic supports based on PSILs for the separation and purification of high value biomolecules, such as proteins and nucleic acids. Additionally, the multi-modal and affinity character of some ILs could represent considerable improvements upon the separation of complex biomolecules. In this field, Alves et al. [86] synthetized a new chromatographic support from Sepharose CL-6B containing a 3-(10-carboxydecyl)-2-methylbenzothiazol-3-ium bromide as a ligand for protein purification. The main goal of this work was to evaluate the individual contribution of the azolium functional group as a constituent part of thiacarbocyanine dyes in an affinity or multi-modal relationship with standard proteins. This important work has shown the potential application of multi-modal IL-based ligands, such as benzothiazolium bromide, compared to its congener cyanine dyes, for the separation and purification of proteins. The PSIL investigated revealed a distinct and useful chromatographic behavior by itself [86]. Recently, in our group, a macroporous chromatographic support (Toyopearl^®^ AF-Epoxy-650M) was functionalized with 1-methyl-3-propylimidazolium chloride to purify nucleic acids [52]. This macroporous resin was functionalized in the same manner as silica supports via halogenated silane groups in a two-step procedure [52]. Furthermore, since this is an activated resin with a high density of epoxy-functionality, the functionalization with ILs can also be performed via the epoxy group through “click” chemistry reactions. The material prepared by Neves et al. [52] was used in preparative liquid chromatography for the efficient separation of three types of nucleic acids, namely ribosomal RNA, small RNA, and genomic DNA, from complex bacterial lysates in just one step. Moreover, since IL acts as a multi-modal ligand, this novel support deserves to be further investigated in the purification of other high-value biomolecules. Table 3 summarizes the works using polymer-supported ILs (PSILs).

PILs have gained substantial interest in the past years, regarding the separation and purification of biomolecules, and to the best of our knowledge, not many studies have been reporting the use of PSILs as chromatographic matrices. Therefore, herein, we also show possible alternative routes for the immobilization of ILs onto agarose-based polymer, such as Sepharose, a widely used chromatographic matrix for the purification of biomolecules.

### 5.1. ILs Immobilization after Co-Polymerization of Vinyl-Functional Polymers

An example of the ILs immobilization after co-polymerization of vinyl-functional polymers was first described by Fontanals et al. [83], where the starting polymer was obtained by polymerization of vinylbenzyl chloride (VBC) and divinylbenzene (DVB) in the presence of toluene and catalyzed by benzoyl peroxide. After the polymerization, the material obtained (VBC-DVB copolymer) was left to react with an excess of N-methylimidazole [83]. Thus, the PSIL could be prepared in a simple nucleophilic substitution reaction between any imidazole groups and a halogenated alkane molecule (R—Cl) present in the functional copolymer (e.g., VBC). Cl anions could be then substituted by other anions, such as trifluoroacetic (CF_3_COO^−^) [83].

A different approach is to use the benzyl group of the copolymer as the nucleophile that is going to react with the added halogenated alkane molecule (e.g., butyl chloride) by an SN reaction and form an alkyl-pyridinium polymer [64].

### 5.2. ILs Immobilization onto Agarose Polymers via Steglich Esterification

The immobilization of a functional IL containing a free carboxyl group, such as 3-(10-carboxydecyl)-2-methylbenzothiazol-3-ium bromide, onto Sepharose can be performed by Steglich esterification, using dicyclohexylcarbodiimide (DCC) as a coupling reagent and 4-dimethylaminopyridine (DMAP) as basic catalyzer. In the work performed by Alves et al. [86], Sepharose CL-6B was left to react with benzothiazolium in the presence of DCC, DMAP, and N,N-dimethylformamide (DMF). In detail, DCC and the carboxylic acid can form an *O*-acylisourea intermediate, holding a similar reactivity with the corresponding carboxylic acid anhydride. Then, the alcohol easily reacts with this activated carboxylic acid to form the desired ester. Hence, the carboxylic group moiety of the benzothiazolium makes this compound reactive and potentially able to bind onto hydroxylic groups of Sepharose (an agarose commercial support) [86].

### 5.3. ILs Immobilization onto Agarose Polymers after Cyanogen Halide Activation

Axéan and Ernback [87] described the activation of Sephadex by means of a cyanogen halide (such as cyanogen bromide, CNBr), which give rise to the formation of imidocarbonates (highly reactive) and carbamic acid (inert) esters on the agarose surface. After this CNBr activation, the ethyl imidocarbonate group on the agarose surface can react with amine groups, such amino-acids or derivatives, in mild pH aqueous solutions [87]. Therefore, this is a possible chemical route for the immobilization of AA-based ILs onto agarose polymers. Furthermore, Bethell et al. [88] described a different method for the activation of Sepharose CL-6B with 1,1-Carbonyldiimidazole (CDI method) in dioxane solution, producing an highly reactive compound at the surface of agarose resin that could react also with primary amine groups of some ILs.

### 5.4. ILs Immobilization onto Agarose Polymers after Epichlorohydrin Activation

Another means for the activation of agarose beads is to introduce an epoxy reactive group at its surface. For the preparation of this support, agarose is suspended in 0.8 M NaOH solution containing NaBH_4_ and an epichlorohydrin solution [89]. Afterwards, an IL containing a nucleophilic group (-NH_2_, -SH, and -OH) could be added to the epoxy-activated support at 25 °C in sodium bicarbonate solution (basic pH environment) with a pH ranging from 8.5 to 11. In addition to this method for epoxy activation, Platis and Labrou [90] described the use of 1,4-butanediol diglycidyl ether (bisoxirane) instead of the epichlorohydrin to activate Sepharose CL-6B in a similar way.

## 6. Conclusions and Future Perspectives

In the past decades, ionic liquids (ILs) have been publicized as green solvents mainly due to their non-volatility. However, not all ILs are safe and nontoxic, and they must be carefully selected. Recently, several works highlighted the potential of more biocompatible and biodegradable ILs as potential alternatives to imidazolium counterparts [36]. For instance, examples of more benign ILs have been reported, such as those composed of cholinium-, glycine-, and glycine-betaine-based cations, combined with anions derived from carboxylic acids [91,92], biological buffers [93], and amino acids [94]. Nevertheless, one of the most relevant properties of ILs in the field of separations is related to their designer solvent ability, this property being transferred to supported ionic liquids applied in liquid chromatography. Despite the fact that the liquid state of ILs is lost when immobilized, their capability to establish a plethora of interactions is kept, allowing them to be used in hydrophilic, hydrophobic, affinity, multi-modal, and ion-exchange chromatography. Due to their advantages, IL-modified materials have been recently synthetized and proven to be an important new type of stationary phases in liquid chromatography, as reviewed here [35].

Herein, we have described different ILs immobilization strategies on typical chromatographic supports, namely silica and polymers, as new alternatives to the more conventional matrices already used in liquid chromatography. Despite their relevant applications summarized here, namely regarding their applicability in analytical field (separation of small molecules by HPLC or SPE), further applications of SILs in separation of high-value biomolecules, such as proteins and nucleic acids with interest in the pharmaceutical, biomedical, and other biochemical industries, are still missing. With this in mind, the advances made on preparative chromatographic matrices by coupling specific ligands allowed a more efficient and specific purification of such complex biomolecules. In this field, the use of amino-acid-based ILs [95] as potential ligands for the selective separation and purification of nucleic acids from complex biological samples plays a particular role, as amino acids have good biocompatibility, are easily available, and are already used as ligands in conventional supports for these applications [28,29]. Amino-acid-based ILs contain multiple functional groups, such as an imidazolium cation, carboxylic, and amino groups, allowing them to be highly effective by exploring their multiple interactions.

Although not investigated to date, it is relevant to highlight the possibility of using ILs as new ligands of monoliths as well. Monolithic columns have the advantages of good permeability, easy preparation, and fast mass transfer. Considering these features, great interest has been shown in the monolithic materials for chromatographic separation of biomolecules, such as nucleic acids [27,29]. Therefore, monolithic-based supported ILs can combine the exceptional advantages of supported ILs with monolithic columns, which can then display higher column efficiency and resolution than conventional ones.

Within biotechnological processes, liquid chromatography is the method of choice for biomolecules purification. However, this industry is highly demanding for novel chromatographic ligands based on multimodal interactions, so that more selective and efficient separations of relevant biomolecules can be reached. From our perspective, ionic liquids as new ligands undoubtedly comprise a potential multimodal behavior when interacting with a variety of analytes, as highlighted in this review, and should be deeply explored in this regard.

## Figures and Tables

**Table 1:** Different types of chromatographic supports used in the purification of various biomolecules.

Bioproduct	Used Matrices	References
Adenoviral vector	Fractogel TMAE	[22]
sc pDNA	Capto^TM^ adhere resin	[23]
Lactoferrin	Sulfanilic acid-modified chitosan mini-spheres	[24]
Nucleic Acids	Sepharose CL-6B treated with 1,4-butanediol diglycidyl ether	[25]
mcDNA	Sephacryl S-1000 SF matrix	[26]
mcDNA	Cadaverine modified monolith	[27]
pre-miRNA-29	L-arginine–Sepharose 4B gel	[28]
sc pDNA	Histidine-agarose, arginine-macroporous, Histidine-monolith	[29]

**Table 2:** SILs preparation by single cation immobilization mechanism used in separation processes.

Type of Ligand	Application	Analytes	Type of Sample	Type of Immobilization	References
C8, C10, Naph, C4-Ph	RPLC	polycyclic aromatic hydrocarbons	mixture solutions	via halogenated silane groups	[62]
Py	AEX-LC	organic compounds/aromatic hydrocarbons; inorganic anions	mixture solutions	via halogenated silane groups (heterogeneous process)	[57]
MIm	AEX-LC	inorganic anions	mixture solutions	via halogenated silane groups (heterogeneous process)	[56]
MPIm, BPIm	RPLC	aromatic carboxylic acids	mixture solutions	via halogenated silane groups (homogeneous process)	[63]
EMIm	SPE	liquiritin and glycyrrhizic acid	licorice extract	via halogenated silane groups (heterogeneous process)	[64]
Im, Mim, EMIm, ImBF4, ImNTf2	LC	xylose and glucose	mixture standard solution and a solution of enzymatically hydrolyzed water	via halogenated silane groups and further modifications	[50]
NIm	HPLC	aromatic organic compounds; alkaloids	mixture solutions	via halogenated silane groups and further modifications	[60]
Im, MIm, EMIm	SPE	Lactic acid	Fermentation broth	via halogenated silane groups (heterogeneous process)	[64]
MIm	RPLC/IEX	proteins	mixture solution and egg white	via halogenated silane groups (heterogeneous process)	[65]
2-MIm, 1-MIm	HILIC/AEX-LC	Sulfonamides; nucleosides/nucleobases; vitamins; saccharides; inorganic anions	mixture solutions	via halogenated silane groups (heterogeneous process)	[61]
MIm	HILIC	Sulfonamides; nucleosides/nucleobases	mixture solutions	via halogenated silane groups (heterogeneous process)	[59]
BIm, NaphIm, AIm	SPE	2,4-dinitrophenol	aqueous solutions	via halogenated silane groups	[66]
C18Im, MIm+C18	RPLC	alkylbenzenes, alkylnaphthalenes and PAHs	mixture solutions	via halogenated silane groups (heterogeneous process)	[67]
P_3_NIm, SP_3_NIm	SPE	2,4-dinitrophenol	aqueous solutions	via halogenated silane group; via thiol-containing silane (“thiol-ene” click reaction)	[68]
SC8ImLac	HILIC/RPLC	PAHs, anilines, and high polar compounds	milk powder, Trichoderma sp. extract	via thiol-containing silane (“thiol-ene” click reaction)	[69]
SNGlu	HILIC/AEX-LC	nucleotides and flavonoids	mixture solutions	via thiol-containing silane (“thiol-ene” click reaction)	[52]
SONIm	HILIC	nucleosides, amino acids, organic acids, flavonoids, etc.	flavonoids mixture, soybean flavonoids, and urine	via thiol-containing silane (“thiol-ene” click reaction)	[70]
SImCalix	RPLC/HILIC/AEX-LC	alkyl benzenes, phenols, nucleosides, and anions	mixture solutions	via thiol-containing silane (“thiol-ene” click reaction)	[71]

Legend: C8: 1-Octynyldimethylchlorosilane, C10: 1,5-Decadiynyldimethylchlorosilane, Naph: β-Naphthyldimethylchlorosilane, C4-Ph: 4-phenyl-l-butyldimethylchlorosilane, Py: Propylpyridinium chloride, MIm: N-methylimidazolium chloride, MPIm: 1-methyl-3-propylimidazolium bromide, BPIm: 1-n-butyl-3-propylimidazolium bromide, EMIm: 2-Ethyl-4-methylimidazolium chloride, Im: imidazolium chloride, ImBF_4_: imidazolium tetrafluoroborate, ImNTf_2_: imidazolium bis(trifluoromethanesulfonyl)imide, NIm: amino-propylimidazolium bromide, 2-MIm: 2-methylimidazolium chloride, 1-MIm: 1-methylimidazolium chloride, BIm: 1-benzylimidazolium chloride, NaphIm: 1-(1-Naphthylmethyl)imidazolium chloride, AIm: N,N-dimethylaminopropylimidazolium chloride, C18Im: 1-octadecylimidazolium chloride, MIm+C18: N-methylimidazolium chloride in a octadecyl silica material, P_3_NIm: 1-alkyl-3-(propyl-3-amino)imidazolium bromide, SP_3_NIm: 1-allyl-3-(propyl-3-amino) imidazolium bromide, SC8ImLac: 1-vinyl-3-octylimidazolium L-lactate, SNGlu: N,N-diallyl-N-methyl-d-glucaminium bromide, SONIm: 1-vinyl-3-(amide)imidazolium bromide, SImCalix: calixarene-based 1-allyl-imidazolium bromide.

**Table 3:** Summary on the PSILs used in separation of bioactive compounds and biomolecules.

Matrix	IL (Ligand)	Application	Samples	Refs.
VBC-DVB copolymer	imidazolium trifluoroacetate	SPE by anion exchange	acidic analytes from real water samples	[83]
PS-PVP copolymer	aminopropyl-imidazolium	SPE	bioactive compounds from *Sophora Flavescens Ait*	[84]
MI-PS-PVP copolymer	imidazolium, methylimidazolium, carboxyl-imidazolium, amino-imidazolium, cyano-imidazolium chloride	SPE	tanshinones from *Salvia miltiorrhiza Bunge*	[85]
PVPB copolymer	alkyl-pyridinium chloride	SPE	liquiritin and glycyrrhizin from Licorice	[46,64]
Sepharose CL-6B	benzothiazolium bromide	multi-modal chromatography	protein solutions of RNase, α-chymotrypsin and BSA	[86]
Toyopearl^®^ AF-Epoxy-650M	1-methyl-3-propylimidazolium	multi-modal chromatography	three types of nucleic acids from complex bacterial lysates	[52]

## Data Availability

Not applicable.
